# Dopamine Receptor Partial Agonists: Do They Differ in Their Clinical Efficacy?

**DOI:** 10.3389/fpsyt.2021.781946

**Published:** 2022-01-25

**Authors:** Pavel Mohr, Jirí Masopust, Miloslav Kopeček

**Affiliations:** ^1^National Institute of Mental Health, Klecany, Czechia; ^2^Faculty of Medicine, Charles University, Prague, Czechia; ^3^Psychiatric Clinic of the University Hospital Hradec Kràlové, Charles University, Hradec Kràlové, Czechia

**Keywords:** antipsychotics, dopamine partial agonists, aripiprazole, brexpiprazole, cariprazine, clinical efficacy

## Abstract

Dopamine receptor partial agonists (DRPAs; aripiprazole, brexpiprazole, and cariprazine) constitute a novel class of antipsychotics. Although they share a similar mechanism of action, DRPAs differ in their pharmacodynamics, pharmacokinetics, drug interactions, or safety and tolerability. The antipsychotic efficacy of all three drugs was established in several placebo-controlled randomized trials (RCTs) in schizophrenia, both acute phase and relapse prevention. In addition, each of the DRPA agents has been tested in other psychiatric disorders, including bipolar disorder or major depression. However, a few studies have examined their comparative clinical efficacy. There are no head-to-head comparisons between aripiprazole, brexpiprazole, or cariprazine. In two acute schizophrenia RCTs of cariprazine and brexpiprazole, aripiprazole was used as an indirect comparator to control for study sensitivity. To assess potential differences in the efficacy of DRPAs, we reviewed data from controlled trials, systematic reviews, and meta-analyses. Our results showed that the acute antipsychotic effects of DRPAs, as measured by the number needed to treat, are comparable. The three agents were superior to placebo in acute treatment, and cariprazine was found to be effective in the reduction of primary negative symptoms of schizophrenia. In the therapy of bipolar disorder, aripiprazole and cariprazine showed antimanic efficacy, cariprazine was also effective in the management of bipolar depression, and aripiprazole was effective for relapse prevention. The addon administration of aripiprazole or brexpiprazole reduced symptoms of major depression. Aripiprazole can control acute agitation associated with psychosis or bipolar disorder; brexpiprazole showed the potential to manage agitation in dementia patients. Aripiprazole has also established evidence of efficacy in children and adolescents and other conditions: OCD, tic disorders, and autism spectrum disorder. Our review of published data suggests that in terms of clinical efficacy, DRPAs are a heterogeneous group, with each drug possessing its own therapeutic benefits.

## Introduction

Antipsychotic drugs represent the mainstay of schizophrenia treatment (1). They all share a common mechanism of action: antagonism at postsynaptic dopamine D_2_ receptors (2). However, one group of antipsychotics, dopamine receptor partial agonists (DRPAs), differs in its effects on the dopamine system. Unlike most of the other antipsychotic agents, DRPAs also possess intrinsic dopamine D_2_/D_3_ agonist activity and act differently (either as agonists or antagonists) in various parts of the brain (3).

At present, three DRPAs have been approved for clinical use: aripiprazole, brexpiprazole, and cariprazine. Comparing their pharmacodynamics, aripiprazole has the highest intrinsic D_2_ activity, and cariprazine has the highest D_3_ activity (Table 1) (4, 5). High intrinsic D_2_ activity can explain activating effects of aripiprazole; high selective affinity for D_3_ receptors of cariprazine is effective on positive, negative, and cognitive symptoms. Moreover, hyperstimulation of D_2_ or D_3_ receptors can cause restlessness, akathisia, agitation, insomnia, nausea, dyspepsia, or rarely compulsive or impulsive behavior (e.g., hypersexuality, compulsive shopping, pathological gambling, and overeating) (4, 6). Brexpiprazole with a lower intrinsic D_2_ and D_3_ has less activating effects and lower risk for akathisia, insomnia, nausea, and dyspepsia.

**Table 1:** Human receptor affinity of dopamine receptor partial agonists (DRPAs) and potential clinical effects (modified from 4, 5).

**Receptor**	**Type of activity**	**Affinity** ***K*****i (nM)** ***in vitro***			**Potential clinical effects**
		**Aripiprazole**	**Brexpiprazole**	**Cariprazine**	
Dopamine D_2_	Partial agonist	0.34	0.30	0.49	Antipsychotic effect, extrapyramidal syndrome (EPS), prolactin elevation,
	*Intrinsic activity*	*60%*	*45%*	*30%*	akathisia, nausea, insomnia, subjective response to treatment
Dopamine D_3_	Partial agonist	0.8	1.1	0.08	Effects on positive and negative symptoms, procognitive effect, EPS,
	*Intrinsic activity*	*28%*	*15%*	*71%*	akathisia
Serotonin 5-HT_1A_	Partial agonist	1.7	0.12	2.6	Antidepressant and anxiolytic effects, procognitive effect, reduction of EPS
	*Intrinsic activity*	*73%*	*60%*	*39%*	
Serotonin 5-HT_2A_	Antagonist	3.4	0.47	19	Reduction of EPS, weight gain
Serotonin 5-HT_2B_	Antagonist	0.36	1.9	0.58	? (unknown)
Serotonin 5-HT_2C_	Antagonist	15	34	134	Weight gain
Serotonin 5-HT_7_	Antagonist	10.3	3.7	111	Antidepressant and procognitive effects
Histamine H_1_	Antagonist	28	19	23	Sedation and weight gain, hypnotic and anxiolytic effects
Adrenergic alpha_1A_	Antagonist	26	3.8	155	Vasodilatation, hypotension, sedation, antihypertensive effects, improvement of prostate hypertrophy, effect on nightmares
Adrenergic alpha_1B_	Antagonist	35	0.17	>155	? (unknown)
Adrenergic alpha_2C_	Antagonist	38	0.59	>155	Antidepressant and prosexual effects
Muscarinic M_1_	Antagonist	>1,000	>1,000	>1,000	Dry mouth, blurred vision, constipation, urinary retention, tachycardia, cognitive impairments, delirium

DRPAs also vary in their relative affinity for other neurotransmitter systems: Brexpiprazole has the highest occupancy of the serotonin 5-HT_1a_ and 5-HT_2a_ receptors, and adrenergic alpha_1a_, alpha_1b_, and alpha_2c_ receptors (4). High affinity for the 5-HT_1a_ and 5-HT_2a_ receptors reduces the risk of extrapyramidal symptoms (EPS) and improves anxiety and depression symptoms. Blockade of the adrenergic alpha_1a_ and alpha_1b_ receptors increases the risk of sedation and hypotension; alpha_2c_ antagonism has antidepressant and prosexual effects. All three drugs have low affinity for serotonin 5-HT_2c_, histamine, and muscarinic receptors, and thus, possess low risk of metabolic side effects, weight gain, and anticholinergic effects (4).

Following oral administration, DRPAs reach maximum serum concentrations within 3–6 h (4). Their respective bioavailability is 87% (oral aripiprazole), 95% (brexpiprazole), 97% (intramuscular aripiprazole), and 52–80% (cariprazine). The elimination half-life is 75 h for aripiprazole (active metabolite dehydro-aripiprazole has 94 h), 91 h for brexpiprazole, and 48–96 h for cariprazine; its active metabolites have half-lives of 30–38 h (desmethyl-cariprazine) and 168–504 h (didesmethyl-cariprazine). DRPAs are mainly metabolized via the hepatic cytochrome isoenzymes CYPD6 and CYP3A4.

Pharmacodynamics and pharmacokinetics predict clinical effects and tolerability (Table 1). Thus, given their variances in pharmacology, it is reasonable to assume that DRPAs differ in their efficacy. There is a paucity of data comparing their antipsychotic effects between each other, the only available (indirect) comparison is based on the number needed to treat (NNT) (4, 7). To assess potential differences in the efficacy of DRPAs further, we reexamined data from randomized controlled trials, systematic reviews, and meta-analyses.

## Methods

All publicly available records on PubMed, Cochrane Library, and ClinicalTrials.gov were searched using the key words “aripiprazole,” “brexpiprazole,” or “cariprazine.” Included were randomized, double-blind trials with neuropsychiatric patients that examined clinical efficacy. Additional entries were obtained through appropriate references; retrieved records were checked for duplicity. In addition to the randomized controlled trials (RCTs) as the primary data source, meta-analyses of DRPAs assessing comparative efficacy (between DRPAs, DRPAs vs. placebo or standard treatment) or generalizing efficacy data were included, where available. If there was more than one meta-analysis examining the same subject, the most inclusive and/or methodologically sound one was used. The results were reviewed by the authors narratively according to the conditions in which the drugs were tested.

## Results

### Clinical Efficacy

A total of 143 randomized, double-blind, placebo- or active-comparator controlled trials of DRPAs were identified as of August 31, 2021. The therapeutic efficacy of aripiprazole, brexpiprazole, and cariprazine was tested in 68 RCTs with schizophrenia (Table 2), 26 RCTs with bipolar disorder, 19 RCTs with major depression (Table 3), and 30 RCTs in other neuropsychiatric disorders (Table 4).

**Table 2:** Randomized double-blind trials of DRPAs in schizophrenia.

**Indication**	**Aripiprazole**	**Brexpiprazole**	**Cariprazine**
Acute treatment (overall symptom change)	**ARI oral** 27 RCTs vs. placebo: SMD = −0.41 (95% CI −0.32, −0.50) Registration studies: NNT = 8 (95% CI 6, 13) 4 RCTs vs. risperidone: n.s. (SMD = −0.07; 95% CI −0.35, −0.20) 4 RCTs vs. olanzapine: n.s. (SMD = −0.15; 95% CI −0,32, 0.02) 1 RCT vs. ziprasidone: n.s. (SMD = −0.16; 95% CI 0.50, 0.18) 3 RCTs vs. haloperidol: n.s. (SMD = 0.00; 95% CI −0.24, 0.23) **ARI LAI** 2 RCTs vs. placebo: ALAI monohydrate: *p* < 0.0001 ALAI lauroxil: *p* < 0.001	6 RCTs vs. placebo: SMD = −0.26 (95% CI −0.12, −0.39) NNT = 7 (95% CI 5, 12)	4 RCTs vs. placebo: SMD = −0.34 (95% CI −0.20, −0.49) NNT = 10 (95% CI 7, 18)
Relapse prevention	**ARI oral** 1 RCT vs. placebo: time to relapse: *p* < 0.001 HR: 0.59 (95% CI 0.35. 0.71) relapses: 34% vs. 57% (*p* < 0.001) 2 RCTs vs. haloperidol: time to relapse: *p* = 0.0001 HR: 0.81 (95% CI 0.57. 1.14) relapses/treatment failures: 17% vs. 21%	1 RCT vs. placebo: time to relapse: *p* < 0.0001 HR: 0.292 (95% CI 0.156. 0.548) impending relapses: 13.5% vs. 38.5% (*p* < 0.0001)	1 RCT vs. placebo: time to relapse: *p* = 0.001 HR: 0.45 (95% CI 0.28, 0.73), NNT = 5 relapses: 24.8% vs. 47.5%
	**ARI LAI** 1 RCT vs. placebo: time to relapse: *p* < 0.0001 HR (PL/ALAI): 5.03 (95% CI 3.15, 8.02) impending relapses: 10.0% vs. 39.6% 2 RCTs vs. ARI oral (= noninferiority)		
Acute agitation	**ARI im** 2 RCTs vs. placebo: ARI 5.25 mg: *p* ≤ 0.05 ARI 9.75 mg: *p* < 0.001 responses: 55% vs. 36%. NNT = 5	n/a	n/a
Children and adolescents	1 RCT vs. placebo: ARI 10 mg: *p* = 0.05; remission NNT = 6 ARI 30 mg: *p* = 0.007; remission NNT = 5	n/a	
Predominant negative symptoms	n/a	n/a	1 RCT vs. risperidone: LSMD in PANSS-FSNS: −1.46 (95% CI −2.39, −0.53), *p* < 0.01 responses: 69% vs. 58%; NNT = 9
Treatment-refractory patients	***Clozapine augmentation*** 7 RCTs vs. placebo: total symptoms: SMD = −0.57 (95% CI −1.02, −0.13)	n/a	n/a

*n/a, no studies available; ARI, aripiprazole; ARI LAI/ALAI, aripiprazole long-acting injectable; ARI im, aripiprazole intramuscular injection; ARI oral, aripiprazole oral formulation; HR, hazard ratio; LSMD, least squares mean difference; NNT, number needed to treat; n.s., no significant difference; PANSS, Positive and Negative Syndrome Scale; PANSS-FSNS, PANSS-factor score for negative symptoms; RCT, randomized controlled trial; PL, placebo; SMD, standardized mean difference*.

**Table 3:** Randomized double-blind trials of DRPAs in mood disorders.

**Indication**	**Aripiprazole**	**Brexpiprazole**	**Cariprazine**
Bipolar disorder *manic/mixed episode*	6 RCTs vs. placebo (monotherapy): responses (3 W): 47.7 vs. 31.4%. NNT = 6 remissions (3 W): 39.6 vs. 32.4%. NNT = 14	2 RCTs vs. placebo: reduction in YMRS: LSMD = 0.14 (95% CI −1.74, 2.03) (n.s.) LSMD = −1.62 (95% CI −3.56, 0.32) (n.s.)	3 RCTs vs. placebo: responses: 57% vs. 36%. OR = 2.30 (95% CI 1.78, 2.98), NNT = 5, *p* < 0.001 remissions: 46% vs. 30%. NNT = 7. *p* < 0.001
Bipolar disorder *depressive episode*	2 RCTs vs. placebo: *negative results*	n/a	4 RCTs vs. placebo: reduction in MADRS: 1.5 mg/day SMD = −0.26 (95% CI −0.49, −0.02) 3 mg/d SMD = −0.21 (95% CI −041, −0.01) remissions: NNT = 10 (1.5 mg); NNT = 14 (3 mg)
Bipolar disorder *maintenance treatment*	**ARI oral** 1 RCT monotherapy vs. placebo: 26 W time to relapse: *p* = 0.020 number of relapses 25 vs. 43% (*p* = 0.013) 100 W time to relapse: *p* = 0.011 1 RCT adjuvant therapy: time to relapse of mania: *p* < 0.01	n/a	1 RCT vs. placebo: results not available
	**ARI LAI** 1 RCT vs. placebo: time to relapse: *p* < 0.0001 number of relapses: 26.5 vs. 51.1% (*p* < 0.0001)		
Bipolar disorder *acute agitation*	**ARI im** 1 RCT vs. placebo responses: 66 vs. 37%. NNT = 3	n/a	n/a
Bipolar disorder *children and adolescents*	4 RCTs vs. placebo: response in acute mania: *p* < 0.01 relapse prevention: *p* < 0.05	n/a	n/a
Unipolar depression *adjunctive treatment*	3 RCTs vs. placebo: reduction in MADRS: −10.3 vs. −6.5; *p* < 0.0001 responses: 36 vs. 19%, NNT = 6 remissions: 24 vs. 12%, NNT = 8	9 RCTs vs. placebo: reduction in MADRS: SMD = −0.20; (95% CI −0.29, −0.11) responses: NNT = 17 remissions: NNT = 25	5 RCTs vs. placebo: 1 positive study (2.5–4/day mg: change in MADRS *p* = 0.01; responses: NNT = 9) 2 negative studies 2 studies: no results available
Unipolar depression *elderly patients*		2 RCTs vs. placebo: well tolerated, no efficacy data	

*n/a, no studies available; ARI, aripiprazole; ARI LAI/ALAI, aripiprazole long-acting injectable; ARI im, aripiprazole intramuscular injection; ARI oral, aripiprazole oral formulation; LSMD, least squares mean difference; MADRS, Montgomery–Asberg Depression Rating Scale; NNT, number needed to treat; n.s., no significant difference; OR, odds ratio; RCT, randomized controlled trial; PL, placebo; SMD, standardized mean difference; W, week; YMRS, Young Mania Rating Scale*.

**Table 4:** Randomized double-blind trials of DRPAs in other indications.

**Indication**	**Aripiprazole**	**Brexpiprazole**	**Cariprazine**
Alzheimer's dementia	***Psychosis*** 3 RCTa vs. placebo: 1 study: NPI-NH *p* = 0.013 2 negative studies ***Agitation*** ARI im (10–15 mg) > PL	***Agitation*** 2 RCTs vs. placebo: efficacy of 2 mg/d (CMAI) SMD = −3.77 (95% CI −7.38, −0.17), *p* = 0.040 SMD = −5.06 (95% CI −8.99, −1.13), *p* = 0.012	n/a
OCD (adjunctive therapy)	2 RCTs vs. placebo: YBOCS, WMD = 7.32 (95% CI −12.99, −1.66)	n/a	n/a
Tic disorder and Tourette syndrome	17 RCTs vs. active treatment vs. PL: SMD = −4.74 (95% CI −8.67, −1.06)	n/a	n/a
Autism spectrum disorder	3 RCTs vs. placebo: responses: RR = 2.08 (95% CI 1.24, 3.46); NNT = 4 (95% CI 2.8, 5.7)	n/a	n/a
Alcohol dependence	1 RCT vs. placebo: Days abstinent: 58.7 vs. 63.3% (n.s.) 1 RCT vs. naltrexone: Number of alcohol-free subjects: 12 vs. 11 (n.s.) number of relapsed subjects: 4 vs. 7 (n.s.)	n/a	n/a

*n/a, no studies available; ARI im, aripiprazole intramuscular injection; CMAI, Cohen–Mansfield Agitation Inventory; NNT, number needed to treat; NPI-NH, Neuropsychiatric Inventory-Nursing Home; n.s., no significant difference; OCD, obsessive-compulsive disorder; PL, placebo; RCT, randomized controlled trial; RR, response rate; SMD, standardized mean difference; WMD, weighted mean difference; YBOCS, Yale–Brown Obsessive Compulsive Scale*.

#### Schizophrenia—Acute Treatment

Aripiprazole was found to be superior to placebo in numerous short-term studies of adult patients with acute schizophrenia. A recent comprehensive meta-analysis comparing the antipsychotic efficacy of 32 oral drugs analyzed a total of 27 placebo- or active-comparator-controlled RCTs with aripiprazole (8). The oral formulation of aripiprazole was significantly more efficacious than placebo in the overall change of symptoms (*n* = 1,926 patients; standardized mean difference (SMD) = −0.41, 95% CI −0.50, −0.32), positive symptoms (*n* = 1,451; SMD = −0.38, 95% CI −0.48, −0.28), negative symptoms (*n* = 1,353; SMD = −0.33, 95% CI −0.41, −0.24), and depressive symptoms (*n* = 150; SMD = −0.40, 95% CI −0.69, −0.10), but not in social functioning (*n* = 50; SMD = −0.23, 95% CI −0.55, 0.09).

Moreover, the antipsychotic efficacy of aripiprazole was compared with risperidone in four RCTs (in two of them, it was used to control for study sensitivity). It was compared with olanzapine in four RCTs, with haloperidol in three placebo-controlled RCTs, and with ziprasidone in one RCT (8). In the pairwise comparisons, aripiprazole was as effective as all active comparators (Table 2).

Aripiprazole was found to be more effective than placebo in the treatment of children and adolescents with schizophrenia (9). The results of a 6-week RCT showed that 10 or 30 mg/day of aripiprazole in patients 13–17 years old improved schizophrenia symptoms, as measured by the PANSS, Clinical Global Improvement (CGI), and Children's Global Assessment Scale. The difference from placebo in the total PANSS score at study end was significant for both doses, 10 mg (*p* = 0.05) and 30 mg (*p* = 0.007).

The efficacy of brexpiprazole 2 and 4 mg in the treatment of acute schizophrenia was established in six RCTs (8). Brexpiprazole was more effective than placebo in overall symptom reduction (*n* = 1.180; SMD = −0.26, 95% CI −0.39, −0.12), improvement of positive symptoms (*n* = 1,180; SMD = −0.17, 95% CI −0.31, −0.04), negative symptoms (*n* = 1,180; SMD = −0.25, 95% CI −0.36, −0.14), depressive symptoms (*n* = 1,090; SMD = −0.16, 95% CI −0.29, −0.03), and social functioning (*n* = 918; SMD = −0.25, 95% CI −0.38, −0.12).

The antipsychotic efficacy of cariprazine has been proven by the results of four, 6-week RCTs in acute schizophrenia (8). Compared with placebo, cariprazine improved significantly more overall symptoms (*n* = 999; SMD = −0.34, 95% CI −0.49, −0.20), positive symptoms (*n* = 999; SMD = −0.30, 95% CI −0.45, −0.16), negative symptoms (*n* = 999; SMD = −0.34, 95% CI −0.44, −0.20), and depressive symptoms (*n* = 305, SMD = −0.36, 95% CI −0.63, −0.09). No data on social functioning of cariprazine were obtainable from acute trials.

#### Schizophrenia—Relapse Prevention

For maintenance treatment and prevention of schizophrenia relapse, oral aripiprazole outperformed the placebo or haloperidol in three long-term trials. In a 26-week study, 310 patients were randomized to aripiprazole 15 mg/day or placebo (10). The time to relapse was significantly longer for aripiprazole than for placebo (*p* < 0.001), and more patients relapsed on placebo (57%) than on aripiprazole (34%). The relative risk of relapse for the aripiprazole group was 0.59 (95% CI 0.35, 0.71; *p* < 0.001), the risk was reduced by 41%. Additionally, 30 mg of aripiprazole daily was as effective as 10 mg/day of haloperidol in two, 1-year RCTs with similar protocols, and the total study sample consisted of 1,294 patients (11). Based on a 30% improvement in the Positive and Negative Syndrome Scale (PANSS) total score maintained for at least 28 days, aripiprazole produced a significantly higher response rate than haloperidol (52 vs. 44%; *p* < 0.003). Time to discontinuation for any reason was significantly greater with aripiprazole than with haloperidol (*p* = 0.0001), more relapses or treatment failures were reported for haloperidol (21%) than aripiprazole (17%). Compared with haloperidol, aripiprazole reduced the risk of relapse by 19% (hazard ratio HR = 0.81, 95% CI 0.57, 1.14).

The long-term efficacy of brexpiprazole in maintenance treatment of schizophrenia was evaluated in a year-long, double-blind trial (12). Patients with acute psychotic symptoms were switched to open-label treatment with brexpiprazole 1–4 mg/day over a period of 1–4 weeks. Patients completing the conversion phase entered a 12- to 36-week stabilization phase. In this phase, patients were titrated to a dose of brexpiprazole (1–4 mg/d). Those who remained stable (*n* = 202) were then randomized to continuation treatment with either their stabilization dose of brexpiprazole or placebo. Compared with placebo, brexpiprazole significantly delayed the time to relapse (*p* < 0.0001) and reduced relapse risk by 71% (HR = 0.29, 95% CI 0.16, 0.55). The proportion of patients meeting the criteria for impending relapse was 13.5% with brexpiprazole and 38.5% with placebo (*p* < 0.0001), the relative risk of relapse for the brexpiprazole group was 0.35.

A 97-week, placebo-controlled, multicenter study assessed efficacy of cariprazine in the long-term maintenance treatment of schizophrenia (13). In the first open phase, during an 8-week run-in period, the patients were given a flexible dose of cariprazine 3–9 mg/daily and then kept on a fixed dose over the course of a 12-week stabilization period. The patients (*n* = 200) were subsequently randomized to a blinded administration of cariprazine (3, 6, or 9 mg/day) or placebo. Double-blind phase lasted from 26 to 72 weeks. The results demonstrated that maintenance treatment with cariprazine was superior to placebo in terms of time to relapse (*p* < 0.001), and the risk of relapse was reduced by 55% (HR = 0.45, 95% CI 0.28, 0.73). Relapse occurred in 24.8% of cariprazine- and 47.5% of placebo-treated patients; the relative risk of relapse for the cariprazine group was 0.52.

#### Schizophrenia—Treatment Resistance and Primary Negative Symptoms

Aripiprazole demonstrated efficacy in augmenting clozapine in treatment-resistant schizophrenia in short- and long-term treatments (14). In a systematic review and meta-analysis of seven placebo-controlled trials (duration from 8 to 24 weeks) with 486 clozapine-refractory patients, addon aripiprazole produced improvement in the total psychotic symptoms (SMD = −0.57, 95% CI −1.02, −0.13) and in negative symptoms in five RCTs (*n* = 328; SMD = −0.33, 95% CI −0.55, −0.11). However, the reduction of positive symptoms was nonsignificant (*n* = 328; SMD = −0.15, 95% CI −0.60, 0.30).

The specific effect of cariprazine on negative symptoms of schizophrenia was investigated in a separate, actively controlled RCT (15). A total of 461 patients with predominant negative symptoms and minimum positive symptoms were randomized to 26 weeks of treatment with either cariprazine (4.5 mg/day) or risperidone (4 mg/day). Cariprazine produced a greater reduction of negative symptoms than risperidone, and the difference was consistently significant from week 14 to the end of the study. The cariprazine-induced change in the PANSS factor score for negative symptoms was −8.90 vs. −7.44 points in the risperidone group; the least squares mean difference (LSMD) was −1.46 (95% CI −2.39, −0.53; *p* = 0.0022), effect size (ES) at 0.31. Cariprazine also outperformed risperidone in the secondary efficacy measure, the Personal and Social Performance Scale, the change of the total score was 14.30 for cariprazine vs. 9.66 for risperidone; (LSMD = 4.63, 95% CI 2.71, 6.56; *p* < 0.0001; ES = 0.48).

#### Bipolar Disorder—Manic Episode

Aripiprazole monotherapy in the management of the acute manic phase of bipolar disorder was tested in seven RCTs, four 3-week and three 12-week studies, with a total of 2,303 patients (16). In all but one trial, aripiprazole was compared with placebo, in two it was compared with haloperidol, and in one it was compared with lithium. Across all studies, aripiprazole achieved significantly higher response and remission rates than placebo. Response was defined as ≥50% improvement in the Young Mania Rating Scale (YMRS) total score, remission as the YMRS total score ≤12. At week 3, the average response rate was 47.70% for aripiprazole, 45.99% for the comparator agents, and 31.40% for placebo. At week 12, the response rate was 59.12% for aripiprazole and 50.63% for the controls. A meta-analysis of the effect sizes (aripiprazole vs. placebo) suggested a pooled d-value equal to 0.34 (95% CI 0.24, 0.44) for YMRS (16). In addition to monotherapy, aripiprazole at 15 and 30 mg/day (*n* = 1,101) was found to be effective for acute mania as an addon therapy to lithium or valproate compared with placebo or haloperidol (17). Aripiprazole demonstrated similar efficacy for improving manic or mixed episodes, psychotic or non-psychotic manic episodes, or rapid cycling.

Two 3-week, placebo-controlled RCTs with brexpiprazole for acute mania were negative (18). In the total sample of 654 patients, brexpiprazole (flexible dosing of 2–4 mg/day, titrated to a maximum of 4 mg/day) failed to separate from the placebo in the reduction of the YMRS score. Response or remission rates were not reported. A gradual improvement of manic symptoms was observed only in a 26-week, open-label extension in the subjects who completed acute studies (*n* = 381): the mean decrease of the YMRS total score was −14.0 (SD 8.9).

A summary analysis of three placebo-controlled studies with cariprazine in the treatment of acute manic or mixed episode included 1,037 patients (19). Trial designs were similar; after a week-long washout period, patients received a 3-week, double-blind treatment. Two studies used a flexible dosing schedule of cariprazine, 3–12 mg daily, and the third included two active groups of 3–6 mg/daily and 6–12 mg daily. The results at week 3, measured by the YMRS score reduction, demonstrated significant efficacy of cariprazine in the management of acute mania compared with placebo in response (57 vs. 36%; NNT = 5) and remission (46 vs. 30%; NNT = 7) rates. Cariprazine was superior to placebo in the effect size for both response rate (ES = 2.31, 95% CI 1.35, 3.95; *p* = 0.021) and remission rate (ES = 2.05, 95% CI 1.61, 2.61; *p* = 0.006) (20). The risk difference (RD) of response rates was 0.204 (95% 0.090, 0.317; *p* = 0.0163; NNT = 5), the RD of remission rates was 0.165 (95% 0.125, 0.206; *p* = 0.003; NNT = 6).

#### Bipolar Disorder—Depressive Episode

Neither aripiprazole monotherapy (5–30 mg/day) nor adjunctive therapy was found to be effective in the treatment of depressive episode of bipolar disorder. Although aripiprazole in two 8-week RCTs (*n* = 749) reduced the severity of depressive symptoms, its efficacy did not separate it from placebo (21). No double-blind controlled studies of brexpiprazole in bipolar depression have been reported.

Cariprazine monotherapy dosed within the range of 0.25 to 3 mg daily was tested in two 6-week and two 8-week, placebo-controlled studies with 1,799 patients (20). The primary results of one 8-week study were negative. In all others, cariprazine at 1.5 or 3.0 mg/day improved symptoms of acute bipolar depression. The effect size of cariprazine at 1.5 mg in the reduction of the Montgomery–Asberg Depression Rating Scale (MADRS) scores was −0.26 (95% CI −0.49, −0.02; *p* = 0.040), and the ES for 3 mg cariprazine was −0.21 (95% CI −0.41, −0.01; *p* = 0.045).

Cariprazine at 1.5 mg/day produced non-significant response rates on MADRS (≥50% reduction of the MADRS score) with an ES of 1.53 (95% CI 0.78, 2.99; *p* = 0.113) and a trend for higher remission rates on MADRS (MADRS score ≤10) with an ES of 1.75 (95% CI 0.96, 3.22; *p* = 0.057). The RD of response rates on MADRS was 0.10 (95% −0.06, 0.26; *p* = 0.112; NNT = 10, the RD of remission rates was 0.10 (95% −0.02, 0.23; *p* = 0.072; NNT = 10). The 3 mg/day of cariprazine yielded a statistically significant ES for response rate according to the MADRS, with an odds ratio (OR) of 1.55 (95% CI 1.11, 2.17; *p* = 0.030) and a remission rate with an OR of 1.53 (95% CI 1.36, 1.72; *p* = 0.043). The RD of response rates according to MADRS was 0.10 (95% 0.02, 0.18; *p* = 0.030; NNT = 10). The RD of remission rates was 0.07 (95% 0.04, 0.11; *p* = 0.01; NNT = 14) (20).

#### Bipolar Disorder—Maintenance Therapy

No DRPA has established efficacy in the prophylaxis of both symptom domains of bipolar disorder. Only aripiprazole was found to be effective in preventing relapse to mania in both monotherapy and in combination with mood stabilizers. Moreover, there is an ongoing trial with cariprazine in the relapse prevention of bipolar disorder, but the results are not available yet (22). No study of brexpiprazole in the maintenance treatment is registered.

In a placebo-controlled trial of 161 patients, aripiprazole doses of 15 and 30 mg daily were effective in delaying the time to manic relapse after 26 and 100 weeks but not in preventing relapse to depression (23, 24). At week 26, aripiprazole-treated patients had significantly fewer relapses (25%) than patients on placebo (43%; *p* = 0.013).

A 52-week study of addon aripiprazole 10–30 mg/day in combination with lithium or valproate demonstrated significantly better efficacy over placebo in the prevention of relapses in patients with mania (*p* < 0.01) but not in subjects with mixed episodes (25). In a randomized sample of 337 patients, there was a significantly greater reduction in the YMRS total score from baseline with aripiprazole in both manic (treatment difference = −3.32, *p* < 0.01) and mixed episode populations (treatment difference = −2.56, *p* = 0.02).

#### Bipolar Disorder—Children and Adolescents

Aripiprazole was tested in several RCTs in children and adolescents with acute bipolar mania and in maintenance therapy. In a 4-week study with 296 patients 10–17 years old, aripiprazole doses of 10 or 30 mg daily reduced the symptoms of mania significantly more than placebo (26). The response (≥50% reduction in the YMRS total score) was achieved in 44.8, 63.6, and 26.1% of subjects in the aripiprazole 10 mg, aripiprazole 30 mg, and placebo groups, respectively (*p* < 0.01 for both doses vs. placebo). The acute phase was followed by a 26-week blinded maintenance phase with 210 study subjects (27). The overall time to all-cause discontinuation was longer for 10 mg/day aripiprazole (15.6 weeks) and 30 mg/day aripiprazole (9.5 weeks) than for placebo (5.3 weeks; both *p* < 0.05 versus placebo). The two aripiprazole doses were significantly superior to placebo in response rates, as well as on all other measures.

Aripiprazole also significantly delayed the time to a new episode (hypomania, mania, mixed state, or continued cycling) in a 72-week, placebo-controlled study of maintenance treatment with 96 bipolar outpatients aged 4–9 years (28). There was a high attrition rate over the first four study weeks: 50% in the aripiprazole group and 90% in the placebo group.

A small 6-week study with 43 patients aged 8–17 years old who suffered from mania comorbid with ADHD found that aripiprazole (mean dose 13.6 ± 5.4 mg daily) was effective at reducing YMRS score vs. placebo (*p* = 0.02; ES = 0.80) (29). Aripiprazole also achieved higher rates of response (*p* = 0.02) and remission (*p* = 0.01).

More recently, a placebo-controlled trial investigated the efficacy of aripiprazole (mean dose 7.1 ± 3.7 mg daily) in a study sample of 59 symptomatic patients aged 5–17 years diagnosed with cyclothymia or bipolar disorder not otherwise specified, with high genetic risk for bipolar disorder (30). At week 12, the subjects responded more to aripiprazole than to placebo (reduction of the YMRS total score: *p* < 0.005).

#### Depressive Disorder—Adjunctive Treatment

The clinical efficacy of addon aripiprazole in the treatment of unipolar depression with insufficient response to antidepressant therapy was established in three, 6-week RCTs with identical designs (31). Patients who failed to improve during the initial 8 weeks of open-label treatment with antidepressants were randomly assigned to adjunctive aripiprazole (2–20 mg/day, based on tolerability) or placebo. The total study sample comprised 746 patients, and in patients with minimal response to previous antidepressant therapy, adjuvant aripiprazole yielded greater improvement in the MADRS total score than placebo (*p* < 0.0001), as well as in response rates (36 vs. 19%; *p* < 0.0001; NNT = 6) and remission rates (24% vs. 12%; *p* < 0.0001; NNT = 8).

A meta-analysis of nine 6- to 24-week RCTs of brexpiprazole for augmentation of antidepressant therapy (total *n* = 3673) showed that brexpiprazole was more effective than placebo for all outcome measures (32). Brexpiprazole was administered in fixed or flexible doses within a range of 0.5–3 mg/day, and one study used quetiapine XR as an active comparator. Treatment with brexpiprazole resulted in a higher response rate (risk ratio RR = 0.93, 95% CI 0.89, 0.97; NNT = 17), remission rate (RR = 0.95, 95% CI 0.93, 0.98; NNT = 25), and reduction of the MADRS total score (SMD = −0.20, 95% CI −0.29, −0.11). Doses beyond 2 mg/day produced a significantly greater RR than doses ≤2 mg/day (RR 0.96 vs. 0.89).

Two small trials (NCT01670279: *n* = 18 and NCT01837797: *n* = 15) evaluated the safety and tolerability of brexpiprazole in elderly patients above 65 years and 70–85 years, respectively (22). Efficacy assessment was planned only for the NCT01837797 study. There were 129 enrolled patients, and only 15 patients were randomized to Period 2 (double-blind addon treatment with brexpiprazole at 1 mg, 3 mg, or placebo) before the study was terminated. Due to the limited number of patients, no efficacy data were collected. Unpublished results of a completed 4-week (plus 2-week follow-up) study of brexpiprazole augmentation (up to 3 mg/day) with intranasal ketamine (40 mg) in 51 depressive patients (NCT03149991) were negative (22).

Published were findings of three 8-week, double-blind trials examining the efficacy of cariprazine as an addon treatment of depression with insufficient response to antidepressants. The first 8-week study with 819 patients who did not respond to a minimum of 6 weeks of antidepressant therapy brought positive results (33). Significant improvement in the MADRS total score vs. placebo was observed for cariprazine doses of 2–4.5 mg/day from week 2 (*p* = 0.011; NNT for response at the endpoint was 9) but not for doses of 1–2 mg/day. The second study with 530 randomized patients failed to demonstrate significant improvement with cariprazine 1.5–4.5 mg daily vs. placebo (34). Similarly, negative results were found in a study with 231 patients that used two cariprazine dosing schedules, 1–2 mg/day or 2–4.5 mg/day (35). Two additional 6-week placebo-controlled RCTs with cariprazine as an adjunctive treatment (1.5–3 mg) to antidepressant treatment in major depression have been completed, but the results are not yet available (22).

#### Other Neuropsychiatric Disorders

The oral formulation of aripiprazole was investigated in three placebo-controlled RCTs in Alzheimer's disease (36). In the management of psychosis, only one of three 10-week studies met the primary efficacy criteria. In a 10-week, placebo-controlled study with 208 outpatients, the mean age was 81.5 years, and aripiprazole (mean end dose of 10.0 mg/day) yielded greater improvement than placebo only in the Brief Psychiatric Rating Scale (BPRS) psychosis (*p* < 0.03) and core (*p* < 0.04) subscales but not in the primary outcome measure, the caregiver assessment Neuropsychiatric Inventory (NPI) psychosis subscale (37). The second study with 256 inpatients used flexible dosing of aripiprazole (2 to 15 mg/day) with a mean aripiprazole dose at the endpoint of 8.6 mg/day (38). There was no significant difference between aripiprazole and placebo in the NPI psychosis subscale, only in several secondary measures, suggesting efficacy on agitation, anxiety, and depression. In the third study, 487 institutionalized nursing home patients were randomized to placebo or fixed doses of aripiprazole at 2, 5, or 10 mg/day (39). Aripiprazole at 10 mg/day produced a statistically significant improvement vs. placebo on the NPI-Nursing Home (NPI-NH) Psychosis subscale score (−6.87 ± 8.6 vs. −5.13 ± 10.0; *p* = 0.013) at week 10, as well as on the secondary measures [BPRS, CGI, Cohen-Mansfield Agitation Inventory (CMAI)].

The clinical efficacy and safety of brexpiprazole in the management of agitation associated with Alzheimer's disease was tested in two placebo-controlled RCTs (40). Both studies were 12-week trials. Study 1 (*n* = 433) used a fixed dose of 2 mg of brexpiprazole daily, and Study 2 (*n* = 270) used a flexible dosing schedule of 0.5–2.0 mg/day. The results showed that agitation, measured by the reduction of CMAI score, was reduced by a dose of 2 mg, but not by lower doses. The dose of 2 mg in Study 1 significantly outperformed placebo (adjusted mean difference −3.77, 95% CI −7.38, −0.17; *p* = 0.040), and *post hoc* analysis of Study 2 also found better efficacy of the maximum dose of 2 mg (−5.06, 95% CI −8.99, −1.13; *p* = 0.012).

Two small double-blind, placebo-controlled studies examined aripiprazole addon to SSRIs in the treatment of obsessive–compulsive disorder (OCD). In the first trial, 40 patients were randomized to 16 weeks of administration of aripiprazole 15 mg/day or placebo (41). Among the 30 completers, adjunctive aripiprazole treatment achieved significantly greater improvement on the Yale–Brown Obsessive Compulsive Scale (Y-BOCS) total score and subscores (obsessions, *p* = 0.007; compulsions, *p* = 0.001; total score, *p* < 0.0001). The second study was a 12-week trial with 39 patients, 32 of whom were evaluable (42). The results were positive, and a fixed aripiprazole dose of 10 mg/daily reduced the Y-BOCS total score significantly more than placebo (*p* < 0.0001). Compared with other antipsychotics in treatment-resistant OCD, addon aripiprazole was less effective than risperidone in a single-blind study (43) and quetiapine in a double-blind RCT (44). However, in a network meta-analysis of 20 RCTs with 790 patients comparing the efficacy and tolerability of antipsychotics in treatment-resistant OCD, aripiprazole did not differ from risperidone, haloperidol, olanzapine, quetiapine, or paliperidone (45). After excluding studies with an overall high risk of bias, only aripiprazole was significantly superior to placebo (mean difference: −7.32, 95% CI −12.99, −1.66).

A meta-analysis of 17 RCTs in the treatment of tic disorders and Tourette syndrome in children and adolescents that comprised 1,305 subjects, found aripiprazole to be as effective as other therapeutics, haloperidol, topiramate, risperidone, or tiapride (46). According to a recent network meta-analysis of 60 RCTs with antipsychotics for tic disorders, aripiprazole was superior to placebo (SMD = −4.74, 95% CI −8.67, −1.06) and tiapride (SMD = −4.27, 95% CI −8.01, −0.58) (47).

The efficacy of aripiprazole in the management of behavioral impairments in children and adolescents with autism spectrum disorders was examined in a meta-analysis of three RCTs that included 408 patients (48). The change in psychiatric symptoms and behavioral disturbances measured by the Aberrant Behavior Checklist showed that compared with placebo, aripiprazole significantly reduced scores across various domains: irritability (weighted mean difference, WMD −5.41, 95% CI −7.58, −3.24), hyperactivity/non-compliance (WMD = −7.68, 95% CI −9.92, −5.45), inappropriate speech impairments (WMD = −1.23, 95% CI −2.08, 0.38), and stereotypic behavior (WMD= −2.04, 95% CI−3.33, −0.74), but not lethargy/social withdrawal. The overall pooled response rate of the aripiprazole-treated group was significantly higher than that of the placebo-treated group, with an RR of 2.08 (95% CI 1.24, 3.46), and the NNT was 4 (95% CI 2.8, 5.7).

Several controlled trials tested adjuvant aripiprazole in substance use disorders and in patients with comorbid substance use and schizophrenia or depression (49, 50). The findings from studies on cocaine or amphetamine/methamphetamine dependence remain inconclusive.

In addition to human laboratory and neuroimaging studies, aripiprazole monotherapy was investigated in several open and controlled clinical trials of the treatment of alcohol dependence (51). In a 12-week, placebo-controlled study with 295 patients, aripiprazole failed to outperform placebo on the primary outcome measure, percentage of days abstinent, and percentage of subjects without a heavy drinking day and the time to first drinking day (52). Aripiprazole (5–15 mg/day) was as effective as naltrexone (50 mg) in a 16-week study of 75 patients in the number of alcohol-free subjects, the number of subjects who relapsed, the mean number of abstinent days, and heavy drinking days (53). Patients treated with aripiprazole remained abstinent for a longer time than those treated with naltrexone, but naltrexone treatment resulted in a greater reduction in craving scores.

#### Other Drug Formulations

Aripiprazole is the only DRPA available in various drug formulations. In addition to oral formulation of tablets, orally disintegrating tablets, oral solution, intramuscular (acute) injections, or long-acting (depot) injections (LAI). In 2017, the US Food and Drug Administration (FDA) approved aripiprazole tablet with ingestible sensor, using a digital ingestion system to monitor whether the medication was taken. Long-acting injectable brexpiprazole is currently under clinical investigation; no efficacy data are accessible (22).

Both formulations of long-acting injectable aripiprazole (ALAI) once-monthly, ALAI monohydrate and ALAI lauroxil, were effective in two 12-week studies of acute schizophrenia exacerbation (54, 55). In a study sample of 340 patients, ALAI monohydrate 400 mg reduced PANSS total score at week 10 significantly more than placebo (mean difference in the least square (LS) change: −15.1, 95% CI −19.4, −10.8; *p* < 0.0001) and the CGI-Severity score (CGI-S) (LS −0.8, 95% CI −1.1, −0.6; *p* < 0.0001) (54). ALAI lauroxil also outperformed placebo in the PANSS total score improvement in a study with 623 patients (55). The difference (LS) in the 441 mg group was −10.9 ± 1.8 (*p* < 0.001) and in the 882 mg group was −11.9 ± 1.8 (*p* < 0.001). The proportion of patients who were very much or much improved on the CGI-Improvement (CGI-I) scale was significantly greater with aripiprazole lauroxil 441 and 882 mg treatments vs. placebo (*p* < 0.001).

Aripiprazole LAI was also tested for schizophrenia relapse prevention. ALAI monohydrate at a dose of 400 mg per 4 weeks was superior to placebo in a 1-year study with 403 patients (56). The trial was terminated prematurely because efficacy was already demonstrated by the preplanned interim analysis. The time to impending relapse was significantly delayed with ALAI treatment compared with placebo in both the interim analysis and the final analysis (*p* < 0.0001). Additionally, ALAI 400 mg once-monthly showed non-inferiority to oral aripiprazole in two long-term trials. In the first study, 662 patients were randomized to 26 weeks of treatment with ALAI or 10–30 mg/day of oral aripiprazole (57), and the second 1-year study with 455 patients used 6–24 mg/day oral aripiprazole (58).

ALAI monohydrate 400 mg once-monthly also showed prophylactic efficacy for bipolar disorder (59, 60). In a 1-year study with 266 patients, ALAI significantly delayed the time to recurrence of any mood episode compared with placebo (hazard ratio HR: 0.45, 95% CI, 0.30, 0.68; *p* < 0.0001) and time to hospitalization (p = 0.0002), with a recurrence rate of 2.3% for the ALAI group vs. 13.5% for placebo (HR = 0.14, 95% CI 0.04, 0.47). ALAI primarily reduced the number of manic episodes (*p* < 0.0001).

A randomized, open-label, rater-blinded study compared head-to-head ALAI monohydrate (400 mg once-monthly) with paliperidone palmitate LAI (PP-LAI; 78–234 mg once-monthly) over the course of 28 weeks of treatment (61). The study sample consisted of 295 schizophrenia patients, and the primary outcome measure was the Heinrichs–Carpenter Quality-of-Life Scale (QLS). The results revealed a statistically significant least squares mean difference between the treatments in the change from baseline to week 28 on the QLS total score [4.67 (95% CI 0.32, 9.02), *p* = 0.036] in favor of ALAI over PP-LAI, suggesting superiority of ALAI on clinician-rated quality of life. Moreover, ALAI was also significantly more efficacious for clinical improvement measured by the change in the CGI-S (*p* < 0.01).

The primary goal of a naturalistic, 1-year randomized trial was to compare clinical efficacy, substance craving intensity, and quality of life between aripiprazole LAI (400 mg once-monthly) and paliperidone LAI (100 mg once-monthly) in 101 patients with psychosis (schizophrenia spectrum or bipolar disorder) and comorbid substance use (62). The results showed that both drugs yielded significant improvements, with a comparable effect in the reduction of psychopathology, but aripiprazole was superior in the improvement of quality of life and craving reduction.

A short-acting intramuscular injectable formulation of aripiprazole was effective at reducing acute agitation associated with psychosis in two placebo-controlled studies with an analogous design (63, 64). Aripiprazole (1–15 mg i.m.), haloperidol, or placebo were administered to a total of 805 acutely psychotic patients diagnosed with schizophrenia, schizoaffective disorder, or schizophreniform disorder. The doses of aripiprazole, 5.25 or 9.75 mg, significantly outperformed placebo and were as effective as an active comparator of a haloperidol intramuscular injection (6.5–7.5 mg/day). Compared with placebo, aripiprazole produced a 1.5- to 2-fold greater reduction in mean PANSS-Excited Component scores (PANSS-EC) 2 h after the first dose (primary endpoint).

An intramuscular injectable formulation of aripiprazole significantly reduced acute agitation associated with manic episode in a single, placebo-controlled trial (65). A study sample of 301 acutely agitated bipolar patients was randomized to 9.75 or 15 mg of aripiprazole injection, lorazepam 2 mg i.m., or placebo. Aripiprazole at doses of 5 mg and higher was superior to placebo and as effective as lorazepam. Improvements in the PANSS-EC scores at 2 h were significantly greater with i.m. aripiprazole (9.75 mg, −8.7; 15 mg, −8.7) and i.m. lorazepam (−9.6) vs. i.m. placebo (−5.8; *p* ≤ 0.001).

Aripiprazole i.m. injection showed efficacy in the reduction of acute agitation in Alzheimer's, vascular, or mixed dementia in a trial with a primary outcome of tolerability and safety (66). In a 24-h study of 129 inpatients, doses of 10 or 15 mg (but not 5 mg) were superior to placebo in the improvement of PANSS-PEC at 30 min and maintained their superiority throughout the 24-h study.

### Comparative Efficacy

None of the double-blind randomized studies in various indications compared directly, head-to-head, efficacy between the reviewed dopamine receptor partial agonists. Three acute schizophrenia trials, two double-blind and one open-label, used aripiprazole as an indirect comparator of the tested DRPAs to control for study sensitivity (22, 67, 68).

The objective of a placebo-controlled, 6-week Phase II RCT (NCT00905307) was to establish the optimal dose of brexpiprazole in acute schizophrenia based on efficacy, safety, and tolerability (22). The results have not been published yet. In a failed study with a sample of 459 patients, active treatment arms (brexpiprazole at doses of 0.25 mg, 1 ± 0.5 mg, 2.5 ± 0.5 mg, 5 ± 0.5 mg, or aripiprazole 15 ± 5 mg daily) did not differ significantly from placebo in any of the efficacy measures.

The acute antipsychotic efficacy of cariprazine (3 or 6 mg/day) and aripiprazole (10 mg/day) was comparable in a randomized trial with 617 schizophrenia patients (67). A statistically significant reduction in the PANSS total score vs. placebo was similar across all three active arms: cariprazine 3 mg (LSMD −6.9, 95% CI −10.1, −1.9), cariprazine 6 mg (LSMD −8.8, 95% CI −12.9, −4.7), and aripiprazole 10 mg (LSMD −7.0, 95% CI −11.0, −2.9).

Aripiprazole as a positive control to confirm assay sensitivity was also included in an open-label randomized study of brexpiprazole in acute schizophrenia (68). The study aim was to explore changes in efficacy, cognitive functioning, and safety over the course of 6 weeks of treatment with flexibly dosed brexpiprazole (target dose 3 mg/day) or aripiprazole (target dose 15 mg/day) monotherapy. A total of 97 schizophrenia patients were randomly (2:1) assigned to a 6-week open treatment. Improvement observed in the PANSS total score was comparable in both treatment arms: the least squares mean improvement observed at week 6 was −22.9 points for brexpiprazole (*p* < 0.0001 vs. baseline) and −19.4 points for aripiprazole (*p* < 0.0001 vs. baseline).

In clinical practice, the number needed to treat (NNT) represents a useful tool for indirect comparison of therapeutic effects measured by categorical outcomes. NNT indicates how many patients need to be treated with a new treatment to see an additional improvement over a comparator, with lower numbers, single digits, indicating clinically relevant differences. Citrome comprehensively analyzed NNT and the number needed to harm for adverse events and likelihood to be helped or harmed of the DRPAs vs. placebo in registration studies (7, 69). The comparative efficacy of DRPAs in adjuvant treatment of major depression measured by NNT can be extracted from a recent meta-analysis (70). NNT for treatment response from pivotal trials in schizophrenia, bipolar disorder, and major depression are summarized in Table 5.

**Table 5:** Comparison of clinical efficacy of DRPAs vs. placebo using number needed to treat for therapeutic response (NNT, 95% CI) in schizophrenia, acute mania (7), bipolar depression (71, 72), and major depression (70).

	**Aripiprazole**	**Brexpiprazole**	**Cariprazine**
Schizophrenia—acute treatment	8 (6, 13)	7 (5, 12)	10 (7, 18)
Schizophrenia—relapse prevention	5^*^	4^*^	5^*^
Bipolar mania	7 (5, 11)	n.s.	5 (4, 18)
Bipolar depression	45 (10, ∞)	–	10 (7, 21)
Major depressive disorder	9 (5, 24)	16 (8, 52)	16 (10, 34)

* *based on a single study; n.s., no significant difference*.

The greatest NNT for therapeutic response in acute schizophrenia (30% or greater reduction in the PANSS total score) compared with placebo was 7 (95% CI 5, 12) in brexpiprazole (2–4 mg/day), followed by 8 (95% CI 6, 13) in aripiprazole (10–30 mg/day), and 10 (95% CI 7, 18) in cariprazine (1.5–6 mg/day) (7). Nevertheless, due to the overlap of 95% confidence intervals (Table 5), the differences cannot be considered significant. In the maintenance treatment of schizophrenia, NNT for the number of relapses vs. placebo is based on a single trial of each DRPA (10, 12, 13). No difference was observed between the drugs: NNT for aripiprazole was 5 (4.3), NNT of impending relapses for brexpiprazole was 4, and the cariprazine NNT was 5 (4.4).

Likewise, no statistically significant differences between the DRPAs in treatment responses can be detected for acute mania or adjunctive treatment of major depression. The NNT for response in bipolar mania (≥50% decrease in the YMRS total score) was 5 (95% CI 4, 18) for cariprazine 3–6 mg/day and 7 (95% CI 5, 11) for aripiprazole 15–30 mg/day (7). In bipolar depression, only cariprazine demonstrated a positive effect, studies with aripiprazole produced negative results, and no studies with brexpiprazole have been reported. The NNT for cariprazine in bipolar depression was 10 (95% CI 7, 12) for response and 11 (95% CI 8, 22) for remission (73). Non-significant NNT (>45) for aripiprazole confirms the superiority of cariprazine for the treatment of bipolar depression (71).

Therapeutic response in major depressive disorder, defined as a ≥50% decrease in the MADRS total score, yielded an NNT of 7 (95% CI 5, 11) for aripiprazole 2–20 mg/day and 11 (95% CI 8, 20) for brexpiprazole (1–3 mg/day) (7). In a meta-analysis where the dose was not specified and the response was not defined, the greatest response was observed for aripiprazole (NNT = 9, 95% CI 5, 24), followed by cariprazine (NNT = 16, 95% CI 8, 52), and brexpiprazole (NNT = 16, 95% CI 10, 34) (70). Despite the numeric differences in NNT, the great variance and overlap of 95% confidence intervals indicate that the DRPAs do not differ significantly in their antidepressant action.

Other options for comparing efficacy provided meta-analyses. A systematic review and network meta-analysis that included 3,925 patients indirectly compared aripiprazole with brexpiprazole in acute schizophrenia therapy (74). The results confirmed that the response rates of both antipsychotics were significantly superior to placebo: aripiprazole RR = 0.84 (95% CI 0.78, 0.92) and brexpiprazole RR = 0.84 (95% CI 0.77, 0.92). There was no statistically significant difference between the drugs in their efficacy: brexpiprazole vs. aripiprazole RR = 1.0 (95% CI 0.88, 1.12).

A large network meta-analysis of 32 oral antipsychotics enabled us to compare the acute antipsychotic efficacy of DRPAs with that of other antipsychotic agents and placebo (8). All three DRPAs were more effective than placebo in the overall change in psychotic symptoms. In a pairwise comparison, aripiprazole vs. placebo SMD = −0.38 (95% CI −0.51, −0.25), brexpiprazole vs. placebo SMD = −0.25 (95% CI −0.39, −0.11), and cariprazine vs. placebo SMD = −0.37 (95% CI −0.53, −0.21), while no analyzable data for mutual pairwise comparison of the DRPAs were available, network meta-analysis did not detect a significant difference between the DRPAs: aripiprazole vs. brexpiprazole SMD = −0.15 (95% CI −0.32, 0.01); aripiprazole vs. cariprazine SMD = −0.07 (95% CI −0.23, 0.10); cariprazine vs. brexpiprazole SMD = −0.09 (95% CI −0.29, 0.11).

For the treatment of acute mania, a network meta-analysis confirmed the superior efficacy of aripiprazole and cariprazine over placebo: aripiprazole SMD = 0.37 (95% CI 0.2, 0.55) and cariprazine SMD = 0.47 (95% CI 0.22, 0.73) (75). There was no difference between the two drugs in the improvement of manic symptoms measured by YMRS reduction (SMD = 0.1, 95% CI −0.21, 0.41).

In a network meta-analysis of the efficacy and tolerability of second-generation antipsychotic monotherapy in acute bipolar depression, the only DRPA more efficacious than placebo was cariprazine; aripiprazole was not separated from placebo (72). The mean change in the MADRS total score for cariprazine was −2.29 (95% CI −3.47, −1.09), and the odds ratio for response (≥50% improvement in the MADRS) was 1.47 (95% CI 1.17, 1.82). However, the difference in response between cariprazine and aripiprazole failed to reach statistical significance on both outcome measures, change in the MADRS score (SMD = −1.21, 95% CI −3.70, −1.29), and the response rate (OR = 1.35, 95% CI 0.90, 1.95).

### Tolerability

DRPAs are generally well tolerated with a favorable safety profile (Table 6) (4, 76). The highest risk of akathisia, dose-dependent, is reported in cariprazine, and the lowest incidence is reported in brexpiprazole. High doses of cariprazine can also induce extrapyramidal symptoms. Brexpiprazole is associated with a medium risk of weight gain. Aripiprazole treatment can cause nausea, less frequent sedation, or pathological gambling or compulsive behavior. A recent review of EudraVigilance data found a higher reporting odds ratio of impulse control symptoms for aripiprazole than for brexpiprazole or cariprazine (77). All DRPAs have a low risk of metabolic and cardiovascular side effects and are considered as prolactin-sparing drugs.

**Table 6:** Relative risk of the common side effects induced by DRPAs (modified from 4, 76).

	**Aripiprazole**	**Brexpiprazole**	**Cariprazine**
Akathisia	+	+/–	+
EPS	+	+	+
Anxiety	++	+	+
Sedation	+	+/–	+/–
Weight gain	+	++	+
Metabolic effects	+	+	+
Hyperprolactinemia	+/–	+	+/–
Nausea/vomiting	+	+/–	+
Insomnia	+	+/–	+/–
Impulse control symptoms/compulsive behaviors	+	+/–	+/–
QTc prolongation	+	+	+
Postural hypotension	+	+	+

*EPS, extrapyramidal symptoms; +/-, very low; +, low; ++, moderate*.

The relationship between efficacy and safety can be assessed indirectly with the measure of likelihood to be helped or harmed (LLH) (78). LLH values of >1.0 signify that benefit (response) is more likely than harm (adverse event). LLH values of ≥10 mean that the response is at least 10 times more likely to occur than the evaluated side effect. In schizophrenia treatment, LLH ≥ 10 was found for akathisia in brexpiprazole (LLH = 16) and for somnolence in cariprazine (LLH = 10) (69). LLH for response vs. akathisia was 3.1 for aripiprazole and 1.5 for cariprazine. The LLH for response vs. somnolence was 2.5 for aripiprazole and 7.1 for brexpiprazole.

## Discussion

In the absence of direct head-to-head studies between DRPAs, their comparative efficacy can only be estimated from a synthesis of available preclinical and clinical data (79). The reviewed results from controlled trials and meta-analyses clearly indicate that DRPAs do not represent a fully homogeneous group and possess different therapeutic benefits. This finding only corroborates the fact that the clinical effects of pharmacological agents are directly linked to their pharmacological profiles (4).

Our review did not reveal any significant differences between DRPAs in their antipsychotic efficacy, treatment of acute schizophrenia, or relapse prevention. For acute mania, aripiprazole doses above 15 mg/day and cariprazine doses greater than 3 mg/day are clinically effective. Data analysis suggested that cariprazine in the treatment of manic episode may have a particular effect on irritability (Table 7). The lack of antimanic efficacy of brexpiprazole in two RCTs can be attributed to the high placebo response, slow titration schedule, or regional differences (significant separation from placebo was observed in the EU but not the US study sites). Thus, more studies are needed (18).

**Table 7:** Overview of the clinical efficacy of dopamine receptor partial agonists (DRPA).

• All DRPAs have evidence of acute antipsychotic efficacy that is comparable: it is lower than in some other AP2G (clozapine, amisulpride, olanzapine, and risperidone)
• All DRPAs have comparable evidence of preventing relapse of schizophrenia
• Aripiprazole (≥15 mg/day) and cariprazine (≥3 mg/day) are effective in acute mania
• Cariprazine (1.5–3 mg/day) is effective in bipolar depression
• Aripiprazole in monotherapy (15–30 mg/day), as addon (10–30 mg/day), or LAI (400 mg/4 weeks) is effective in maintenance treatment of bipolar disorder and for preventing relapse to mania
• Aripiprazole (5–15 mg/day) and brexpiprazole (≥2 mg/day) are effective as adjunctive treatment for major depression with insufficient response
• Cariprazine (4.5 mg/day) is effective in treatment of primary, predominant negative symptoms of schizophrenia
• Aripiprazole i.m. injection (9.75 mg/day) is effective for acute agitation in psychosis or bipolar disorder
• Brexpiprazole (2 mg/day) has potential efficacy in the management of acute agitation in Alzheimer's dementia
• Aripiprazole has evidence of efficacy in other indications: treatment of schizophrenia and bipolar disorder in children and adolescents (10–17 years), clozapine augmentation in refractory schizophrenia, adjunctive treatment of obsessive–compulsive disorder (OCD), tic disorders, and autism spectrum disorders

In the treatment of bipolar depression, only cariprazine (1.5–3 mg/day) yielded positive results, aripiprazole studies were negative, and no data for brexpiprazole were available. Aripiprazole monotherapy or adjuvant treatment is effective as maintenance treatment of bipolar disorder, only in preventing relapse to mania. The results of a cariprazine trial in the relapse prevention of bipolar disorder are not available yet; no studies have examined the prophylactic efficacy of brexpiprazole.

Aripiprazole (5–15 mg/day) and brexpiprazole (≥2 mg/day) were found to be effective in augmenting treatment-resistant major depression. *Post hoc* analysis suggested that adjuvant brexpiprazole in depressive disorder produces more anxiolytic and sedative effects. The potential therapeutic benefit of addon cariprazine in unipolar depression reported in a meta-analysis (with equivocal results from individual trials) needs to be further corroborated (70).

Additionally, published data suggest that brexpiprazole is expected to have better efficacy than aripiprazole on cognitive deficit in schizophrenia; cariprazine may specifically improve negative symptoms of schizophrenia. Enhancement of higher cerebral functions by partial dopamine agonism can be explained by the increased dopaminergic transmission in the prefrontal cortex. Analogously, the reduction of negative symptoms by lower doses (100–200 mg/day) of the D_2_/D_3_ receptor antagonist amisulpride is ascribed to the fact that at lower concentrations, presynaptic actions prevail and ensure that dopamine is more released at the synapse (80). Efficacy of amisulpride and cariprazine for predominant negative symptoms was also confirmed in a meta-analysis (81).

The first approved DRPA was aripiprazole (launched in the United States in 2002), and it has been used for the longest time period with extensive clinical experience available. Unlike brexpiprazole or cariprazine, aripiprazole was also tested in numerous other indications and special populations: children and adolescents, dementia patients, OCD patients, patients with tic disorders, patients with autism spectrum disorder, clozapine augmentation, and patients with substance use disorders. There are currently several ongoing trials with brexpiprazole and cariprazine in the treatment of other psychiatric conditions (e.g., attention deficit hyperactivity disorder, generalized anxiety disorder, post-traumatic stress disorder, borderline personality disorder, autism spectrum disorder, Alzheimer's dementia, alcohol use, etc.) and more studies in different indications and populations can be expected. Besides long-term availability, more data are available for aripiprazole due to the studies with various drug formulations. Thus, a reliable comparison between DRPAs can be made only for their oral formulas in few indications: schizophrenia, bipolar disorder, and major depression.

It should be noted that the evidence from clinical trials is not fully reflected in approved indications listed in the summary of product characteristics. Moreover, US and EU regulations differ. The European Medical Agency approved all three DRPAs for the treatment of schizophrenia in adults, aripiprazole for schizophrenia in adolescents 15 years of age and older, treatment of bipolar disorder, manic and mixed episodes (adults and adolescents above 13 years), monotherapy or adjuvant treatment in the maintenance treatment of bipolar disorder, and prevention of relapse to mania. The FDA additionally approved aripiprazole as an adjunctive therapy of major depressive disorder in adults, treatment of children and adolescents with acute mania, either as monotherapy or addon therapy to lithium or valproate, treatment of Tourette syndrome in children, irritability associated with autism spectrum disorder, brexpiprazole as an adjunctive therapy of major depression, and cariprazine for acute treatment of adults with manic, mixed, and depressive episodes of bipolar disorder.

Our review is the first comprehensive comparison of the clinical efficacy of partial dopamine agonists across various psychiatric disorders. All available studies, reviews, and meta-analyses, including unpublished records, were analyzed. The findings, summarized in Table 7, can provide useful guidance for clinicians in psychiatric practice. The results were reviewed qualitatively; heterogeneity of the data and the lack of studies in some indications did not allow more detailed statistics beyond NNT. Moreover, due to the primary focus on efficacy and space limitations, we provide only a brief overview and a general comparison of side effects, without specific details for different conditions or patient populations.

While our review focused solely on double-blind, controlled studies, we should be aware of their shortcomings, especially the limited generalizability of the results. Subject selection for RCTs, especially placebo-controlled RCTs, is biased and not representative of the general psychiatric population (82). To fully assess the efficacy and safety of any treatment, real-world data are needed. Finally, efficacy is just one of the factors in the selection of treatment. Although we exclusively examined differences in the therapeutic effects, choosing a drug for a specific patient should always be individualized, balancing risks vs. benefits, considering not only efficacy but also drug tolerability, safety profile, and preferences of the patients.

## Conclusions

The clinical efficacy of aripiprazole, brexpiprazole, and cariprazine in the treatment of various psychiatric disorders has been confirmed in numerous RCTs. Although there are no direct head-to-head comparisons between them, indirect appraisals indicate that there are clinically meaningful differences in their effects that can be attributed to their specific pharmacological profiles. More RCTs with brexpiprazole and cariprazine for various indications are needed, as well as head-to-head studies examining the variances of DRPAs in their efficacy and safety.

## Author Contributions

PM, JM, and MK contributed equally to the study design, data collection, and interpretation of the findings. PM drafted the original manuscript. JM and MK reviewed and edited the paper. All authors contributed to the article and approved the submitted version.

## Funding

The work was supported by the research projects of Charles University in Prague PROGRES Q35 (Third Faculty of Medicine Prague), PROGRES Q40 (Faculty of Medicine Hradec Králové), and the Czech Ministry of Health MH CZ - DRO (UHHK, 00179906).

## Conflict of Interest

PM has been a consultant and received honoraria and/or speaker fees from Angelini Pharma, Janssen-Cilag, Gedeon Richter, Lundbeck, and Viatris (Mylan). JM has been a consultant and received honoraria and/or speaker fees from Angelini Pharma, Janssen-Cilag, Gedeon Richter, Lundbeck, and PharmaSwiss. MK has been a consultant and received honoraria and/or speaker fees from Angelini Pharma, Janssen-Cilag, Gedeon Richter, and Lundbeck.

## Publisher's Note

All claims expressed in this article are solely those of the authors and do not necessarily represent those of their affiliated organizations, or those of the publisher, the editors and the reviewers. Any product that may be evaluated in this article, or claim that may be made by its manufacturer, is not guaranteed or endorsed by the publisher.
